# Primary Ciliary Dyskinesia—Current Diagnostic and Therapeutic Approach

**DOI:** 10.3390/jcm14196808

**Published:** 2025-09-26

**Authors:** Joanna Wrona, Zuzanna Krupa, Marta Zawadzka, Julia Rydzek, Karolina Dorobisz, Julia Bania

**Affiliations:** 1 Faculty of Medicine, Wroclaw Medical University, 50-367 Wroclaw, Poland; zuzanna.krupa@student.umw.edu.pl (Z.K.); marta.zawadzka@student.umw.edu.pl (M.Z.); julia.rydzek@student.umw.edu.pl (J.R.); j.bania@student.umw.edu.pl (J.B.); 2 Department of Otolaryngology, Head and Neck Surgery, Wroclaw Medical University, Borowska 213, 50-556 Wroclaw, Poland; karolina.dorobisz@umw.edu.pl

**Keywords:** cilia, respiratory failure, PICADAR, NA-CDCF test, rhDNase, respiratory physiotherapy, dyskinesia, situs inversus, ultrastructural defects

## Abstract

Primary ciliary dyskinesia (PCD) is a rare, inherited disease with a complex genetic etiology, leading to ciliary dysfunction and impaired mucociliary clearance. This paper presents the current state of knowledge regarding the clinical presentation, diagnostic approaches, and therapeutic strategies in PCD. The role of genetic testing, ultrastructural analysis of cilia, and modern methods such as high-speed video microscopy (HSVA), nasal nitric oxide (nNO) measurement, and immunofluorescence is discussed. The importance of a multi-step diagnostic process is emphasized, given the absence of a single test with both high sensitivity and specificity. Current treatment options—including respiratory physiotherapy, infection management, and control of ENT symptoms—are reviewed, alongside new experimental approaches such as gene and mRNA therapies. This paper highlights the need for early diagnosis and comprehensive, interdisciplinary care for patients with PCD.

## 1. Introduction

Cilia are microscopic microtubule-based projections whose coordinated beating clears the respiratory tract of mucus and pathogens and facilitates gamete transport. Dysfunction of these organelles results in recurrent infections, lung damage, and fertility problems [1,2,3].

Primary ciliary dyskinesia (PCD) is a rare disorder, inherited predominantly in an autosomal recessive manner, caused by mutations in more than 50 genes encoding ciliary proteins [4,5,6]. Impaired mucociliary clearance leads to recurrent respiratory tract infections, chronic rhinosinusitis, otitis media, bronchiectasis, and infertility; symptoms typically present in the neonatal period, including wet cough, nasal discharge, and respiratory distress [5,7,8,9,10]. Approximately half of patients exhibit laterality defects, such as situs inversus or heterotaxy [11].

The prevalence of PCD is estimated at 1:7500–1:20,000 live births, although the true rate is likely higher due to diagnostic challenges [12]. There is no single confirmatory test; diagnosis relies on a combination of nasal nitric oxide (nNO) measurement, high-speed video microscopy analysis of ciliary motion (HSVA), transmission electron microscopy (TEM), and genetic testing, each with limited sensitivity and accessibility [13,14].

Among children, typical features include daily wet cough before 6 months of age, chronic nasal congestion, recurrent otitis media with hearing impairment (≈75% of cases), and early-onset bronchiectasis; over 80% of neonates with PCD require respiratory support within the first day of life [1,4,15]. Many also experience sleep disturbances related to airway obstruction [16].

Early diagnosis and multidisciplinary management—including airway clearance physiotherapy, infection control, otolaryngological care, and pulmonary monitoring—improve respiratory function, hearing, and overall quality of life [6,9]. Therefore, in any child presenting with at least two of the following features—neonatal respiratory distress in a term infant, laterality defect, daily wet cough since infancy, or persistent non-seasonal rhinitis—prompt evaluation for PCD is warranted [4,17].

PCD remains a challenging diagnosis, often delayed due to its nonspecific clinical presentation and the limited availability of specialized testing. In the absence of a gold standard, diagnosis requires a composite approach incorporating microscopic assessment, nasal nitric oxide measurement, ciliary beat pattern analysis, and extensive genetic testing capable of detecting mutations in more than 50 known PCD-associated genes [18,19].

## 2. Methodology

This paper is a narrative review, informed by a structured literature search. The databases PubMed, Embase, and Scopus were searched using the following keywords: “cilia”, “respiratory failure”, “PICADAR”, “North American Criteria Defined Clinical Features (NA-CDCF)”, “rhDNase”, “respiratory physiotherapy”, “CRISPR/Cas9”, “dyskinesia”, “situs inversus”, “ultrastructural defects”, “Kartagener syndrome”. These terms were combined with the Boolean operators AND and OR to ensure a comprehensive search strategy.

The selection process was descriptive rather than systematic, and no PRISMA flowchart was applied. Titles, abstracts, and full texts were screened, and the most relevant studies were included based on their relevance to the aims of this review. A total of 95 publications were incorporated, covering original research articles, narrative reviews, systematic reviews, and books. Only articles published in English were considered.

The collected material was analyzed narratively and synthesized to present the current state of knowledge on the genetic basis, diagnostic methods, and therapeutic strategies in primary ciliary dyskinesia.

## 3. Genetics

PCD is a disease with a highly complex genetic basis; to date, mutations in more than 40–50 genes have been identified as causative [18,20,21,22]. This number continues to grow as ongoing genetic research and the identification of new cases uncover additional disease-associated genes, such as *LRRC56* [23]. PCD is most often passed down through autosomal recessive inheritance, where genetic mutations disrupt the proteins that are essential for cilia structure, movement, and control [24]. The structure of motile cilia is composed of several distinct elements. The axoneme displays a typical structural pattern, with nine doublets of microtubules (each consisting of an A and a B-tubule) positioned in a circular formation surrounding a central pair of single microtubules. The A-tubule is associated with both inner dynein arms (IDA) and outer dynein arms (ODA). The peripheral microtubule doublets are interconnected by nexin links. Additionally, radial spokes connect the peripheral doublets to the centrally located microtubule pair (C1 and C2) [25].

Patients with PCD may have various ultrastructural defects of cilia, such as outer dynein arm (ODA) defects, inner dynein arm (IDA) defects, microtubule disorganisation (MTD) and central pair of microtubules (CP) defects. Different types of these defects are associated with mutations in specific genes, which affect the course and severity of the disease [22,24,25,26,27,28] (see Table 1).

Mutations affecting the motor protein dynein are one of the identified causes of PCD. Mutations in the *DNAI1* gene, responsible for producing an intermediate dynein chain, can lead to a reduced length or complete absence of the ODA. This structural defect impairs ciliary function and increases susceptibility to conditions like chronic sinusitis, middle ear infections, bronchitis, and sometimes situs inversus. A similar impact is observed with changes in the *DNAI2* gene, which also encodes an intermediate dynein chain. What is more, mutations in *DNAH5*, a gene that partly encodes the dynein heavy chain, can compromise both the integrity and functionality of the ODA, ultimately affecting ciliary movement [25]. Notably, *DNAH5* is the most frequently mutated locus and the leading genetic cause of PCD [18]. *DNAH5* and *DNAI1* mutations cause isolated defects in the outer dynein arms, which are usually associated with a milder course of the disease [29,30]. Another gene encoding the dynein heavy chain is *DNAH11*. Mutation in this locus also results in ODA defects, leading to clinical manifestations typical of PCD [25,30]. Mutations in this gene are often associated with relatively preserved lung function [18,21]. However, alterations in *DNAH11* do not alter the ultrastructure of cilia, but rather impair their motility [24,25]. Available data indicate that dynein protein defects disrupt the structure of the dynein arms, which in turn leads to immotility or hypokinesia of the cilia [24,25].

Genes such as *CCDC114* and *CCDC151*, which encode proteins involved in ODA docking, have also been implicated in PCD [24]. The primary function of the additional ciliary protein encoded by the *LRRC50* gene is the attachment of dynein. Therefore, mutations in this gene result in the inability to anchor dynein arms to the A-tubule, leading to the absence of both ODA and IDA [25].

Several other genes are involved in the structural integrity of the axoneme. Mutations in the *CCDC39* and *CCDC40* genes lead to defects in the IDA and MTD. The products of these genes are responsible for the proper arrangement of radial spokes, which in turn ensures the maintenance of appropriate spacing between the peripheral microtubule doublets [24]. The mutations result in a more severe course of the disease and poorer lung function. Children with these mutations have more changes in chest CT scans and a greater tendency to bronchiectasis than children with ODA defects [29,30]. In addition, IDA + MTD defects may be associated with mutations in other genes, such as *C11orf70* (*CFAP300*), which lead to abnormalities in dynein transport and assembly [26]. What is more, mutations *CCDC39*, *CCDC40*, and *C11orf70* disrupt the assembly and transport of dynein arms, leading to complete or partial ciliary immotility [26,29]. Additionally, a mutation in *C11orf70* (*CFAP300*) leads to disorders in the transport and assembly of dynein in cilia [19,20,26].

The *HYDIN* gene encodes a protein localized to the C2 microtubule of the CP. Mutations in this gene result in the absence of central pair projection proteins associated with the C2 microtubule. Furthermore, radial spoke proteins are encoded by genes such as *RSPH9* and *RSPH4A* and mutations in these genes disrupt central pair formation. This structural defect leads to an abnormal, swirling pattern of ciliary beating [25]. These types of mutations do not carry a risk of situs inversus, as the nodal cilia in the embryo naturally lack a CP in their architecture [24,25].

As noted earlier, mutations in *DNAH11* do not alter the ultrastructure of cilia. This phenomenon is not unique to *DNAH11*, as similar observations have been made for mutations in *CCDC164*, *CCDC65* and *RSPH1*, which likewise do not cause detectable structural abnormalities [24,25]. Additionally, some patients present with a reduced number of motile cilia on their epithelial cells, resulting in clinical features that closely mimic those of classical PCD. This distinct phenotype has been linked to mutations in the *CCNO* and *MCIDAS* genes [24].

Missense mutations, frameshift mutations and mutations leading to loss of protein function predominate in PCD [21,31]. Patients may have mutations in more than one gene (e.g., double or triple heterozygotes), which further complicates the clinical picture [20,31].

## 4. Diagnostics

To date, no test has been developed that would constitute the gold standard for diagnosing PCD, so diagnosis of this condition is based on a combination of observation of the typical clinical phenotype and the results of specialized tests [32]. In the past, screening tests used the saccharin test, which assessed mucociliary clearance in the nose by measuring the time it took to taste saccharin placed in tablet form on the inferior turbinate. This method is no longer recommended due to the need for close cooperation with the patient and the difficulty in determining reliable reference values [33].

Genetic testing is challenging due to the large number and size of disease-causing genes [34]. Due to high genetic and ultrastructural heterogeneity, accurate identification of the defect and gene requires advanced molecular and ultrastructural testing [29]. Thanks to next-generation sequencing, it is possible to detect mutations in approximately 70% of PCD cases [21]. Advances in genetics allow for a better understanding of the mechanisms of the disease, more accurate diagnosis, and identification of new genes associated with PCD [22]. Modern genetic panels and whole exome or genome sequencing allow the diagnosis of PCD to be confirmed in 70–94% of patients with symptoms suggestive of the disease, even when other tests (e.g., electron microscopy) are inconclusive or normal [5,35,36,37,38]. The diagnosis can be made by confirming a biallelic mutation in a known gene [39].

Genetic testing enables earlier diagnosis, which is particularly important in children and in populations with a high degree of relatedness, where rapid identification of familial mutations allows for the implementation of appropriate treatment and genetic counseling [38]. Next-generation sequencing allows the detection of both known and new mutations, as well as rare types of PCD that may not be visible in standard ciliary function tests [18,19,35]. Genetic testing allows specific mutations to be linked to disease severity and progression, which may be important for individualizing treatment and prognosis [18]. In situations where access to specialized ciliary function testing is limited, genetic testing may be the primary diagnostic tool [35]. However, genetic testing cannot rule out the disease due to the fact that approximately 35% of the genes responsible for the development of PCD are not yet known [39].

TEM is a test used to detect ultrastructural defects in cilia, the most common of which is the absence or shortening of the outer dynein arms. It is also possible to demonstrate a number of other, less common abnormalities affecting both the outer and inner dynein arms, as well as the central apparatus of the cilium [40]. The challenge with TEM is to obtain a technically satisfactory biopsy sample containing a sufficient number of cilia, so this method requires appropriate skills and a specialized approach [34]. TEM identifies cilia ultrastructural defects in only about 70–79% of patients with primary ciliary dyskinesia (PCD). This means that in the remaining 21–30% of patients, TEM may have normal cilia ultrastructure despite the presence of functional and molecular abnormalities characteristic of PCD [41,42,43].

Predictive tests have also been developed to aid in the decision to refer a patient for specialized testing, the most popular of which are the Primary Ciliary Dyskinesia Rule (PICADAR) and the North American Criteria Defined Clinical Features (NA-CDCF) [44]. The PICADAR test is used in children with chronic wet cough, and points are awarded in seven clinical categories. It has been shown that patients with a test score of ≥10 points have a more than 90% probability of a positive PCD test result [32].

Currently, the diagnosis of PCD is based primarily on the detection of a biallelic mutation in a known gene during genotyping and/or the presence of ciliary ultrastructural defects in TEM. Genetic testing and TEM may be non-diagnostic in up to 30% of cases, resulting in a lack of a clear diagnosis regimen and the need to combine several diagnostic methods [37].

Three-dimensional electron tomography is an advanced ultrastructural imaging technique that enables detailed, three-dimensional analysis of the architecture of cilia in respiratory epithelial cells. This method is an extension of classical TEM, offering significantly higher precision and the ability to detect subtle morphological anomalies that may remain invisible in standard two-dimensional images. Three-dimensional electron tomography involves obtaining a series of images of the same sample fragment from different angles and then reconstructing them computationally into a three-dimensional model. This allows for detailed spatial and quantitative analysis of ultrastructural elements of cilia, such as dynein arms (external and internal), nexin junctions and radial rays. This technique is particularly useful in cases where conventional TEM does not reveal any abnormalities, which is true for up to 30% of patients with PCD, especially those with mutations in the *DNAH11* gene, characterised by a ‘normal ultrastructure’ phenotype. Normal ciliary ultrastructure includes a characteristic microtubule arrangement (9 + 2 or 9 + 0), the presence of dynein arms, radial rays, a transition zone, and a ciliary membrane. Studies using 3D electron tomography have shown a more than 25% reduction in the volume of proximal external dynein arms in patients with *DNAH11* mutations, which was not possible to observe using conventional TEM. Such observations provide new data on the molecular mechanisms underlying PCD and support the phenotypic classification of the disease. An advantage of 3D electron tomography is that it can be used on previously collected and preserved diagnostic samples, which facilitates the integration of this method into existing clinical procedures. Due to its very high resolution and the possibility of three-dimensional spatial analysis, this technique is particularly recommended in the diagnosis of PCD cases with a normal TEM image or in situations where new, previously undescribed genetic variants are suspected. Currently, three-dimensional electron tomography is considered one of the most advanced tools in the diagnosis of complex PCD phenotypes, constituting an important complement to molecular studies and classical ultrastructural methods [45,46]

Studies have repeatedly shown low levels of nNO in patients with PCD, making this test suitable for use as a screening test. However, it is worth noting that nNO levels are also reduced in other respiratory diseases, such as cystic fibrosis or chronic rhinosinusitis, and therefore cannot be used on its own as a definitive confirmation of PCD [47]. The test is suitable for children aged 5 and older due to the need for cooperation—the resistance exhalation technique is recommended, which ensures palatopharyngeal closure and thus prevents contamination of the nasal gas with air from the lower respiratory tract. In older, cooperative children, chemiluminescence analyzers are most commonly used [48]. Technically, it is possible to adapt the nNO sampling method to younger children, but in practice, cut-off values are difficult to determine at this age, and nNO concentrations typically reach low values in infants, increasing with age [49].

High-speed video analysis (HSVA) is a pivotal technique for assessing ciliary function in the diagnosis of PCD. HSVA involves recording the movement of respiratory cilia at high frame rates (typically 400–500 frames per second), enabling detailed evaluation of both ciliary beat frequency (CBF) and ciliary beat pattern (CBP) [29,50,51]. The analysis can be performed manually or with specialized software, which tracks the ciliary tip across frames to generate objective measurements of oscillation and movement patterns [50,52]. HSVA provides rapid results, which is particularly valuable in clinical settings where other diagnostic methods, such as TEM or genetic testing, may take weeks [51,53]. The method demonstrates high sensitivity and specificity for PCD diagnosis—up to 96–100% sensitivity and 91–96% specificity when both CBF and CBP are evaluated together [51]. Repeating HSVA on three separate samples further increases diagnostic specificity compared to a single test [54]. Interobserver agreement is high when standardized protocols are used, though the need for further standardization in sample preparation and interpretation is emphasized [51,52,53]. CBPs observed via HSVA are classified as normal or abnormal. In healthy individuals, cilia exhibit a planar, coordinated beating motion with a frequency typically between 6.3–9.0 Hz at 32 °C [29]. In PCD, a range of abnormal patterns is seen: complete immotility (often with combined outer and inner dynein arm defects), stiff or low-amplitude beating (inner dynein arm or *CCDC39/CCDC40* mutations), rotational or gyrating motion (central complex defects, e.g., *RSPH1/RSPH4A* mutations), or hyperkinetic but ineffective beating (e.g., *DNAH11* mutations) [29,50,55,56]. Some PCD variants show normal or even increased CBF but with abnormal patterns, highlighting the importance of assessing both frequency and pattern [29,55,56].

An effective imaging method for assessing early changes in the airways and lung parenchymal abnormalities in PCD is high-resolution computed tomography (HRCT), in which significant changes include bronchial wall dilation and thickening, excessive mucus accumulation, lung tissue infiltration, ground-glass opacities, or areas of reduced transparency forming a mosaic pattern. This test allows for accurate localization of lesions and assessment of PCD severity, but its usefulness is limited by the need to accumulate radiation doses associated with periodic monitoring of disease progression [33].

Immunofluorescence is an advanced diagnostic technique used in the identification of PCD, enabling the detection and assessment of the location of key ciliary proteins in respiratory epithelial cells. Biological material for analysis is usually obtained by nasal brushing, after which the cells are fixed and treated with specific fluorochrome-labelled antibodies. These antibodies have a high affinity for specific ciliary components such as *DNAH5*, *DNAH11*, *DNALI1*, *GAS8*, *RSPH4A* and *RSPH9*. Microscopic imaging using fluorescence allows for the assessment of the presence and correct distribution of the above-mentioned proteins in the ciliary structure. The absence of a fluorescent signal or its abnormal location suggests the presence of a structural defect, which may be associated with specific pathogenic variants in the genes responsible for cilia function. This technique allows the detection of both classic ultrastructural abnormalities and subtle disorders that may remain invisible in standard electron microscopy, e.g., in the case of mutations affecting the location of radial spokes or dynein arms.

The advantages of immunofluorescence include relatively short waiting times for results (1–14 days), low costs and availability in histopathology laboratories. The high sensitivity and specificity of the method, especially when using a panel of antibodies covering different proteins, makes it a valuable diagnostic tool. However, it should be emphasised that this technique does not allow for the identification of all cases of PCD, as some mutations do not affect the expression or localisation of the detected proteins, and therefore a normal result does not rule out the presence of the disease.

Immunofluorescence is particularly useful in cases with ambiguous electron microscopy images or inconclusive genetic test results, especially in the context of diagnosing radial spoke defects. Currently, this technique is recommended as an integral part of a comprehensive PCD diagnostic algorithm, complementing ultrastructural analysis, ciliary motility assessment, and molecular testing [57,58,59].

Airway epithelial cell cultures used in PCD diagnostics can be divided into two types: native material (ex vivo, e.g., nasal epithelial biopsies) and in vitro cultures (e.g., air–liquid interface (ALI), organoids), which allow controlled assessment of ciliary function [60,61].

The ALI culture is used in studies of PCD to assess ciliary function under controlled in vitro conditions. Basal cells isolated from the nasal epithelium of PCD patients and healthy donors are first cultured under submerged conditions and then differentiated in ALI cultures, in which the apical surface of the cells is exposed to air while the basolateral surface remains in contact with medium. Under ALI conditions, the ciliated epithelium develops cilia, as confirmed by positive β-tubulin staining, and video microscopy analysis enables quantitative assessment of CBF and ciliary beat pattern CBP. In patients with loss-of-function mutations in the *CFAP300* gene, ALI cultures exhibited complete ciliary immotility, with CBF values corresponding only to background levels, in contrast to the regularly beating cilia observed in healthy donor cultures. Immunostaining revealed the absence of *CFAP300* expression in patient-derived cells, whereas the protein was present in wild-type controls. ALI cultures therefore allow the assessment of ciliary function while preserving the mutation-associated phenotype, eliminating the influence of secondary ciliary dyskinesia present in ex vivo samples, and enabling the integration of functional, molecular, and immunofluorescence analyses in the diagnosis of PCD [62].

Other methods in vitro cultures include human airway organoids (AOs). AOs derived from patients with PCD provide a functional model for studying this genetic disorder. Organoids were obtained from non-invasively collected inferior turbinate brushings from patients and healthy donors and subsequently cultured under conditions that allow long-term expansion. After initial growth, AOs were differentiated toward ciliated cells using ciliary medium (CilM), which inhibits Notch signaling and activates BMP, leading to an increased number of ciliated cells at the expense of secretory cells. Differentiated AOs from PCD patients exhibited mutation-specific ciliary motility defects and ultrastructural abnormalities, which in some cases remained undetectable using standard microscopy. Variation in the number of ciliated cells was also observed between patients and organoid batches, highlighting the individual disease phenotype. These AOs enable precise monitoring of both functional and ultrastructural ciliary phenotypes and provide material for genetic editing, allowing for the correction of patient-specific mutations [63].

Three-dimensional explant spheroids (3D-E) derived from nasal epithelium represent a minimally invasive ex vivo model for studying ciliary function in patients with PCD. Cells obtained via nasal brush biopsy spontaneously form spheroids while retaining terminally differentiated cilia, allowing assessment of CBF and CBP within 1–5 days. 3D-E spheroids effectively distinguish PCD patients from healthy controls, showing reduced CBF and abnormal CBP, with a high success rate (82%) and preserved ciliary function for several days. This model partially reflects the in vivo environment and provides a rapid diagnostic and research tool in the context of PCD [64].

Conditional Reprogramming Culture (CRC) is an advanced technique for culturing airway epithelial cells, which is increasingly used in the diagnosis of PCD. This method is based on co-culturing epithelial cells with inactivated fibroblasts and using a Rho kinase inhibitor (Y-27632), which enables their intensive proliferation and long-term maintenance in a state that allows differentiation into ciliated cells. CRC technology allows large quantities of highly differentiated ciliated cells to be obtained even from small samples of biological material, such as nasal swabs or bronchial biopsies. This is particularly important in the paediatric population and in diagnostically difficult cases where material availability is limited [65,66].

In the context of PCD diagnostics, CRC enables the production of homogeneous layers of ciliated cells, which can then be analysed for functionality, e.g., HSVA, ultrastructural analysis (TEM) and the expression and localisation of ciliary proteins (immunofluorescence techniques). In vitro culture eliminates the influence of environmental factors, such as infections or inflammatory processes, which can secondarily disrupt the morphology and function of cilia in vivo, thus minimising the risk of misinterpretation of results. Another advantage of the CRC method is the possibility of long-term storage of material (biobanking), repeatability of analyses and the use of the obtained cells for research into new targeted therapies, which supports the development of a personalised medicine approach in the context of PCD [65,66].

This technique is characterised by very high efficiency (over 90% of cultures are successful) and high yield and quality of the cells obtained, which retain their full morphology and ciliary function, as confirmed by both motility and protein expression studies. Due to the above properties, CRC is a particularly valuable diagnostic tool in situations where other techniques, such as electron microscopy or genetic analysis, do not allow for an unambiguous diagnosis of the disease or when there is a need to obtain more material for functional and molecular studies [65,66].

## 5. Treatment

The current treatment of PCD focuses on preventing and managing the complications of this disorder [67].

The treatment of PCD largely relies on regular monitoring for respiratory infections through sputum cultures and upper airway swabs to identify pathogens, as well as the use of antibiotics to treat acute exacerbations [68]. Antibiotic treatment in patients with PCD should be initiated when pathogens such as Pseudomonas aeruginosa or MRSA are detected [69,70].

In patients with PCD, *P. aeruginosa* is a major opportunistic pathogen frequently involved in chronic lower respiratory tract infections. Infections with this bacterial species are associated with a deterioration in the clinical condition of patients. Research findings suggest that EDTA may serve as an adjuvant in the treatment of chronic infections caused by *P. aeruginosa* by exerting antimicrobial effects and reducing the production of key bacterial virulence factors [71].

In the case of exacerbations, a 14–21-day course of antibiotic therapy is recommended, based on bacterial susceptibility and the patient’s medical history [72]. In clinical practice, however, there is no universally accepted definition of PCD exacerbations. They are typically defined by the presence of at least three out of seven symptoms, such as increased cough, changes in sputum characteristics, shortness of breath, or fever [73].

The BESTCILIA study—a multicenter, randomized, double-blind, placebo-controlled trial conducted across six European PCD treatment centers—investigated the effect of a 6-month azithromycin therapy on the frequency of exacerbations in 90 patients with PCD. The study demonstrated that maintenance therapy with azithromycin is a viable option for patients with frequent exacerbations, potentially reducing the need for additional antibiotic treatments and helping to prevent irreversible lung damage [74].

Treatment options aimed at improving airway clearance in PCD include the use of mucolytics, hyperosmolar agents, and physiotherapy involving airway clearance techniques [75].

N-acetylcysteine (NAC) is a synthetic derivative of the endogenous amino acid L-cysteine. NAC breaks the disulfide bonds in highly cross-linked mucins, thereby reducing mucus viscosity. It also exhibits anti-inflammatory and antioxidant properties [76]. When administered by inhalation, due to its pH-lowering effect, it may be poorly tolerated as it can cause coughing and bronchospasm, whereas its mucolytic effect when given orally is limited [77]. In patients with COPD and bronchiectasis, long-term use of NAC has shown benefits, although studies in patients with PCD have not confirmed a clear improvement in lung function [78].

Hyperosmolar agents, such as hypertonic saline (HS), facilitate airway clearance by hydrating mucus and stimulating coughing [79,80]. The first-ever prospective, randomized, double-blind study evaluated the effect of a 3-month treatment with 7% HS nebulization compared to 0.9% saline in 22 adult patients with PCD. The study measured quality of life (QoL) and lung function. Improvement was observed only in the subjective perception of health in favor of HS. However, the small sample size (22 patients) meant the study lacked sufficient statistical power to detect differences in key functional parameters, which classifies these data as low-level evidence [81].

However, there are isolated reports in the literature suggesting the potential effectiveness of dornase alfa in some patients. A case was described of a 14-year-old girl with PCD who experienced improvement in respiratory symptoms and a 20% increase in Forced Expiratory Volume in one second (FEV1) after 4 weeks of rhDNase treatment, which was sustained for 4 months [82]. A case has also been reported of two siblings with PCD, aged 6 and 17, who started treatment with rhDNase. Both showed improvement in respiratory symptoms and lung function, while discontinuation of the treatment led to a decline in lung function [83]. These cases suggest that rhDNase may have a beneficial effect in some patients with PCD; however, the evidence comes from single case reports and therefore constitutes low-level evidence, highlighting the need for randomized clinical trials (RCTs) to confirm the efficacy and safety of this therapy in this patient population [75].

Inhaled bronchodilator therapy should not be given to all patients with PCD; currently, it is recommended only for those with PCD who also have coexisting asthma and wheezing [68]. A case is described of a patient with Kartagener syndrome and concomitant allergic bronchopulmonary aspergillosis (ABPA) who was treated with inhaled fluticasone and formoterol combination therapy, which improved asthma symptom control and reduced bronchial hyperresponsiveness, while confirming the need for an individualised approach to the use of bronchodilators in patients with PCD and concomitant asthma [84].

In PCD, respiratory physiotherapy is a key component of treatment aimed at airway clearance. Although physical exercise and inhaled beta2-agonists, such as salbutamol, can help dilate the bronchi and thus facilitate mucus removal, their actual impact on the airways in PCD had not been clearly defined previously. A study comparing children with PCD and healthy children showed that lung function parameters—FEV1, Forced Vital Capacity (FVC), and Peak Expiratory Flow Rate (PEFR)—were significantly reduced in the PCD group from the outset. Physical exercise caused a significant increase in PEFR in children with PCD, greater than that observed after salbutamol inhalation. These results suggest that in children with PCD, exercise is a more effective method of bronchodilation than beta2-agonists. Exercise may promote deep breathing and coughing, which supports mucus clearance [85].

Other measures used to improve mucus clearance in patients with PCD include PEP devices, high-frequency oscillation vests (vest therapy), manual chest physiotherapy, postural drainage, and autogenic drainage [39].

New experimental therapeutic approaches for treating PCD are based on the inhalation delivery of DNAI1 mRNA using lipid nanoparticles (LNPs). A study demonstrated that delivering DNAI1 mRNA encapsulated in LNPs to human bronchial epithelial cells (hBEC) in culture and to the lungs of non-human primates resulted in effective expression of the exogenous DNAI1 protein, its integration into the axoneme, and restoration of ciliary motility. The SORT LNP technology enables efficient and targeted delivery of mRNA to specific cells, while inhalation therapy ensures protein translation and its sustained presence for up to 24 days after a single dose. These findings represent a significant step toward developing disease-modifying therapies for patients with PCD, especially those with mutations in the *DNAI1* gene, and open the possibility of extending this method to other genetic defects causing PCD. However, it should be noted that these are preliminary results with a low level of evidence [86].

Another promising treatment for PCD is gene therapy; however, it should be noted that, similar to the above approach, the results are preliminary and based on low-level evidence. In recent years, therapy using adeno-associated virus (AAV) vectors has shown encouraging results. AAV vectors are characterized by low immunogenicity and a broad ability to infect various cell types, including respiratory ciliated epithelial cells. Unfortunately, they have a limited capacity, which poses a challenge when delivering large genes responsible for PCD, such as *DNAH5*, whose sequence far exceeds AAV’s carrying capacity. Additionally, neutralizing antibodies develop in the body, making repeated dosing difficult.

An alternative are lentiviral vectors, which can carry larger DNA fragments and stably integrate into the host genome, ensuring long-term expression of the therapeutic gene. These vectors can be specifically modified to target airway epithelial cells. Although their immunogenicity is lower than that of AAV vectors, repeated therapy is often required.

There is also growing interest in gene-editing technologies like CRISPR/Cas9 in PCD gene therapy, which allow precise correction of mutations without the need to introduce entire DNA sequences [87].

Studies have shown that the mitochondrial protein NDUFAF2, encoded by nuclear DNA, plays a key role in ciliogenesis through its interaction with the centriole protein ARMC9. The function of NDUFAF2 is closely dependent on the proper NAD^+^/NADH ratio, and its deficiency leads to impaired docking of membrane vesicles and destabilization of the transition zone during ciliogenesis. In cellular and animal models, overexpression of NDUFAF2 restored normal mitochondrial function and ciliary structure, suggesting a potential application of gene or protein therapy for defects related to *ARMC9* and similar mutations. The authors also highlight the therapeutic potential of supplements that raise NAD^+^ levels, such as nicotinamide riboside and vitamin B3, to support mitochondrial function and ciliogenesis. Additionally, they propose that autophagy dysregulation may play a significant role in the pathogenesis of ciliopathies, making its modulation a possible therapeutic target. These findings reveal an important link between mitochondrial function and ciliary structure, opening new avenues for the development of targeted molecular therapies in PCD and other ciliary dysfunction disorders [88].

In patients with PCD, otolaryngological care plays an important role in managing common symptoms such as chronic rhinitis, sinusitis, and otitis media with effusion (OME). OME is a leading cause of acquired conductive hearing loss in children and can result in speech development delays; therefore, the goal of treatment is to improve hearing and prevent complications. In children with PCD, treating OME with ventilation tubes can improve hearing, but it often leads to chronic ear discharge and persistent infections [89].

Additionally, according to the EPOS guidelines, PCD is classified as a cause of secondary chronic rhinosinusitis (SCRS). SCRS is classified based on anatomical location, dominant endotype and phenotype (see Table 2).

According to the EPOS guidelines, SCRS in children is defined by the presence of two or more of the following symptoms, with at least one being nasal discharge or nasal obstruction: nasal obstruction, nasal discharge (anterior or posterior), facial pain/pressure, or cough lasting for 12 weeks or more.

EPOS 2020 recommends saline irrigation in the management of SCRS in children and does not recommend adding antibiotics to the saline solution. Oral or intravenous antibiotic therapy is also considered unjustified in children. Intranasal corticosteroids (INCS) are recommended instead.

There is no evidence supporting the effectiveness of adjunctive therapies such as antihistamines, leukotriene receptor antagonists, decongestants, or mucolytics, and therefore, they are not recommended. The exception is their use in coexisting conditions, such as allergic rhinitis or gastroesophageal reflux disease (GERD).

Surgical intervention should be considered in patients who fail conservative treatment, as well as in rare cases of complicated acute sinusitis. Adenoidectomy is the simplest and safest procedure and should be the first surgery performed in younger children with SCRS.

Functional endoscopic sinus surgery (FESS) is a safe and effective treatment for older children and can be used as the initial procedure or after adenoidectomy failure. The decision to perform FESS should take into account disease severity, the child’s age, and any comorbidities [90].

A case of three children with PCD was reported, who presented with recurrent pneumonia and chronic sinusitis. All children underwent FESS, and two also received pressure equalization tubes. Postoperative follow-up showed significant improvement in symptoms, a reduced number of hospitalizations, and a decreased need for pharmacological treatment, confirming the safety and efficacy of FESS in children with severe sinus disease associated with PCD [91].

The treatment methods for CRS in children recommended by the EPOS 2020 guidelines, along with the evidence category and a brief recommendation, are presented in the table below (see Table 3).

In a study involving 24 patients, endoscopic sinus surgery (ESS) was performed in combination with adjuvant therapy, which included nasal saline irrigation, intranasal glucocorticosteroids, and a two-week course of systemic antibiotic therapy. In cases where *Pseudomonas aeruginosa* was present, intranasal colistin was added for 6 months. Twelve months after ESS, improvement in chronic sinusitis symptoms and stabilization of lung function were observed. In some patients, ESS helped prevent the recurrence of *P. aeruginosa* infections in the lungs. These results suggest that comprehensive treatment of sinusitis in patients with PCD may be important not only for sinus symptoms but also for controlling lower respiratory tract infections [92].

## 6. Discussion

Awareness of the rare ciliary disorder known as PCD is steadily increasing; however, diagnosis is still often delayed due to nonspecific clinical symptoms and limited access to advanced diagnostic tests [1,2,3].

Diagnosis requires combining clinical suspicion (e.g., PICADAR, NA-CDCF) with specialized tests—nNO, HSVA, TEM, immunofluorescence, and genetic testing. No single method suffices; multimodal approaches, including CRC and 3D electron tomography, are especially valuable in inconclusive cases or when ultrastructural defects are absent, as in *DNAH11* mutations [36,40,44,48].

Mutations in *DNAH5*, *DNAI1*, *DNAH11*, *CCDC39* and *CCDC40* correlate with specific ultrastructural defects, for example: *CCDC39/40* variants linked to more severe disease and earlier bronchiectasis [18,21,26,67].

Treatment relies on airway clearance techniques and antibiotic therapy; however, optimal regimens are not clearly established. Long-term use of azithromycin reduces the number of exacerbations, but data on its long-term safety and efficacy are lacking. Similarly, the role of mucolytics, hypertonic saline, and rhDNase remains unclear, with available evidence limited to single studies and case reports [67,75,76,81].

Management of ENT symptoms, such as chronic sinusitis or otitis media with effusion, includes intranasal corticosteroids and saline irrigations, with surgery (e.g., adenoidectomy or FESS) considered individually in refractory cases [93,94].

New therapeutic strategies, such as mRNA therapies, AAV vectors, and CRISPR/Cas9, offer potential for causal treatment but remain at an early research stage. Additionally, observations on the role of mitochondrial dysfunction and NAD^+^ metabolism in ciliogenesis suggest possible novel therapeutic avenues [89].

Ultimately, PCD management must be multidisciplinary, combining pulmonology, ENT, genetics, physiotherapy, and psychosocial support. Patient advocacy groups play a vital role in education, emotional support, and care coordination. Continued research into genetic mechanisms, early diagnostics, and innovative therapies will be key to improving outcomes and quality of life for individuals with PCD [86,87].

## 7. Conclusions

Primary ciliary dyskinesia is a rare disorder characterized by high genetic and phenotypic heterogeneity, requiring a multi-step diagnostic algorithm that integrates molecular, ultrastructural, and functional methods. The absence of a single test with high sensitivity and specificity leads to significant diagnostic delays, highlighting the necessity of early case identification and the implementation of targeted therapeutic interventions. Current treatment strategies focus on the prevention and management of respiratory tract infections, improvement of mucociliary clearance, and management of otolaryngological complications; however, the development of gene and mRNA-based therapies offers promising prospects for causal treatment. Optimal patient care requires close collaboration within a multidisciplinary team and the provision of comprehensive educational and psychosocial support, which may significantly improve quality of life and prognosis.

## Figures and Tables

**Table 1:** PCD genes associated with ultrastructural phenotypes of cilia’s axoneme.

Place of Ultrastructural Defect	Mutated Genes
ODA	*DNAH5*, *DNAI1*, *DNAI2*, *DNAL1*, *CCDC114*, *CCDC151*, *ARMC4*, *TXNDC3*, *TTC25*
ODA + IDA	*DNAAF1*, *DNAAF2*, *DNAAF3*, *HEATR2*, *LRRC50*, *DYX1C1*, *ZMYND10 SPAG1*, *CCDC103*, *C21orf59*, *C11orf70*, *PIH1D3*, *LRRC6*
IDA	*KTU*
MTD	*CCDC39* *, *CCDC40* *, *GAS8* *, *RASPH9* ^#^, *RASPH4A* ^#^
CP	*HYDIN*

*—Besides MTD, mutation is the gene also affects the ultrastructure of IDA; ^#^—Besides MTD mutation is the gene also affects the ultrastructure of CP; ODA—Outer dynein arms, IDA—Inner dynein arms, MTD—Microtubule disorganization, CP—central pair of microtubules.

**Table 2:** Classification of secondary chronic rhinosinusitis (SCRS) based on EPOS 2020.

Anatomical Location	Dominant Endotype	Phenotype
Localized (unilateral)	Localized changes	Odontogenic Fungal ball Tumor
Generalized (bilateral)	Mechanical	PCD CF
	Inflammatory	GPA EGPA
	Immunological	Selective Immunodeficiency

PCD—Primary ciliary dyskinesia, CF—Cystic fibrosis, GPA—Granulomatosis with polyangiitis, EGPA—Eosinophilic granulomatosis with polyangiitis.

**Table 3:** Treatment methods for chronic rhinosinusitis (CRS) in children according to EPOS 2020.

Treatment	Evidence Category	Brief Recommendation
Antibiotic therapy	1b (−)	No evidence supporting efficacy of short or long-term therapy in children with CRS
Intranasal corticosteroids	5	Recommended due to anti-inflammatory effects and safety in children
Systemic corticosteroids	1b (+)	More effective than placebo in treating CRS, but with risk of adverse effects
Saline irrigation	1b (+)	Effective and safe method used in children with CRS
Adenoidectomy	4	Effective in younger children with CRS, especially when conservative treatment fails
FESS	4	Safe and effective in older children with refractory CRS or after adenoidectomy

CRS—Chronic rhinosinusitis, FESS—Functional Endoscopic Sinus Surgery. 1b (+)—High-quality evidence (e.g., from randomized clinical trials) supporting efficacy (marked with a plus). 1b (−)—High-quality evidence but lack of efficacy (marked with a minus). 4—Lower quality evidence based on expert opinions or case analyses. 5—Very low-quality evidence, often based on expert consensus or uncontrolled data.

## Abbreviations

The following abbreviations are used in this manuscript:

PCD	Primary ciliary dyskinesia
nNO	Nasal nitric oxide
HSVA	High-Speed Video Microscopy Analysis
TEM	Transmission Electron Microscopy
ODA	Outer dynein arms
IDA	Inner dynein arms
MTD	Microtubule disorganisation
CP	Central Pair of Microtubules
PICADAR	Primary Ciliary Dyskinesia Rule
NA-CDC	North American Criteria Defined Clinical Features
CBF	Ciliary beat frequency
CBP	Ciliary beat pattern
HRCT	High-resolution computed tomography
ALI	Air–liquid interface
aOS	Human airway organoids
CilM	Cells using ciliary medium
3D-E	Three-dimensional explant spheroids
CRC	Conditional Reprogramming Culture
NAC	N-acetylcysteine
HS	Hypertonic saline
ABPA	Allergic bronchopulmonary aspergillosis
FESS	Functional Endoscopic Sinus Surgery
ESS	Endoscopic sinus surgery
OME	Otitis media with effusion
CRS	Chronic rhinosinusitis
SRCS	Secondary chronic rhinosinusitis
COPD	Chronic obstructive pulmonary disease
MRSA	Methicillin-resistant Staphylococcus aureus
CF	Cystic fibrosis
GPA	Granulomatosis with polyangiitis
EGPA	Eosinophilic granulomatosis with polyangiitis
FEV1	Forced expiratory volume in one second
FVC	Forced vital capacity
PEFR	Peak expiratory flow rate
LNP	Lipid nanoparticles
AAV	Adeno-associated virus
